# Slow progression of pediatric HIV associates with early CD8^+^ T cell PD-1 expression and a stem-like phenotype

**DOI:** 10.1172/jci.insight.156049

**Published:** 2023-02-08

**Authors:** Vinicius Vieira, Nicholas Lim, Alveera Singh, Ellen Leitman, Reena Dsouza, Emily Adland, Maximilian Muenchhoff, Julia Roider, Miguel Marin Lopez, Julieta Carabelli, Jennifer Giandhari, Andreas Groll, Pieter Jooste, Julia G. Prado, Christina Thobakgale, Krista Dong, Photini Kiepiela, Andrew J. Prendergast, Gareth Tudor-Williams, John Frater, Bruce D. Walker, Thumbi Ndung’u, Veron Ramsuran, Alasdair Leslie, Henrik N. Kløverpris, Philip Goulder

**Affiliations:** 1 Department of Paediatrics, University of Oxford, Oxford, United Kingdom.; 2 Africa Health Research Institute, Durban, South Africa.; 3 Max von Pettenkofer-Institute, Department of Virology, Ludwig-Maximilians-University, Munich, Germany.; 4 German Center for Infection Research, Munich, Germany.; 5 Department of Infectious Diseases, Ludwig-Maximilians-University, Munich, Germany.; 6 IrsiCaixa AIDS Research Institute, Badalona, Spain.; 7 KwaZulu-Natal Research Innovation and Sequencing Platform, Nelson R. Mandela School of Medicine, University of KwaZulu-Natal, Durban, South Africa.; 8 Department of Statistics, TU Dortmund University, Dortmund, Germany.; 9 Department of Paediatrics, Kimberley Hospital, Kimberley, South Africa.; 10 Germans Trias i Pujol Research Institute, Badalona, Spain; Universitat Autònoma de Barcelona, Cerdanyola del Vallès, Spain.; 11 Faculty of Health Sciences, Centre for HIV and STIs, National Institute for Communicable Diseases, University of the Witwatersrand, Johannesburg, South Africa.; 12 HIV Pathogenesis Programme, Doris Duke Medical Research Institute, University of KwaZulu-Natal, Durban, South Africa.; 13 Ragon Institute of MGH, MIT and Harvard, Cambridge, Massachusetts, USA.; 14 South African Medical Research Council, Durban, South Africa.; 15 Wits Health Consortium, Johannesburg, South Africa.; 16 Blizard Institute, Queen Mary University of London, London, United Kingdom.; 17 Zvitambo Institute for Maternal and Child Health Research, Harare, Zimbabwe.; 18 Centre for Paediatrics and Child Health, Imperial College London, London, United Kingdom.; 19 Nuffield Department of Medicine, University of Oxford, Oxford, United Kingdom.; 20 Oxford NIHR Biomedical Research Centre, Oxford, United Kingdom.; 21 Division of Infection and Immunity, University College London, London, United Kingdom.; 22 School of Laboratory Medicine and Medical Sciences, University of KwaZulu-Natal, Durban, South Africa.; 23 Centre for the AIDS Programme of Research in South Africa, Durban, South Africa.; 24 Department of Immunology and Microbiology, University of Copenhagen, Copenhagen, Denmark.

**Keywords:** AIDS/HIV, Immunology, Adaptive immunity, T cells

## Abstract

HIV nonprogression despite persistent viremia is rare among adults who are naive to antiretroviral therapy (ART) but relatively common among ART-naive children. Previous studies indicate that ART-naive pediatric slow progressors (PSPs) adopt immune evasion strategies similar to those described in natural hosts of SIV. However, the mechanisms underlying this immunophenotype are not well understood. In a cohort of early-treated infants who underwent analytical treatment interruption (ATI) after 12 months of ART, expression of PD-1 on CD8^+^ T cells immediately before ATI was the main predictor of slow progression during ATI. PD-1^+^CD8^+^ T cell frequency was also negatively correlated with CCR5 and HLA-DR expression on CD4^+^ T cells and predicted stronger HIV-specific T lymphocyte responses. In the CD8^+^ T cell compartment of PSPs, we identified an enrichment of stem-like TCF-1^+^PD-1^+^ memory cells, whereas pediatric progressors and viremic adults had a terminally exhausted PD-1^+^CD39^+^ population. TCF-1^+^PD-1^+^ expression on CD8^+^ T cells was associated with higher proliferative activity and stronger Gag-specific effector functionality. These data prompted the hypothesis that the proliferative burst potential of stem-like HIV-specific cytotoxic cells could be exploited in therapeutic strategies to boost the antiviral response and facilitate remission in infants who received early ART with a preserved and nonexhausted T cell compartment.

## Introduction

Children with HIV who are naive to antiretroviral therapy (ART) progress faster to AIDS compared with ART-naive adults (1). In contrast to adults, the immune response in infants favors Th2-polarizing cytokines, limiting their capacity to mount an effective adaptive response against intracellular pathogens such as HIV (2–4). Nonetheless, approximately 10% of ART-naive children with HIV reach the age of 5 years with normal CD4^+^ T cell count and function despite persistent viremia (5). Pediatric slow progressors (PSPs) are therefore much more prevalent than the adult counterpart, adult viremic nonprogressors, who are exceptionally rare (6–9). Both PSPs and adult viremic nonprogressors maintain normal CD4^+^ T cell counts in the absence of ART by a mechanism similar to that described in natural SIV hosts (6, 10, 11) that can control the inflammation and immune activation observed during acute SIV infection through immunoregulatory pathways, including upregulation of the inhibitory receptor PD-1 on CD8^+^ T cells located in the lymph nodes (12, 13). However, the mechanisms leading to this clinical slow-progression phenotype are not fully elucidated in humans.

The low level of immune activation despite ongoing viral replication is a key feature of slow clinical progression and appears to be more compatible with the tolerogenic and Th2-bias immune response observed in early life than with the aggressive Th1-driven immune response observed in adults (14). A highly potent cytotoxic T lymphocyte (CTL) response seen in adults can lead to better viral control, but it is also associated with higher levels of immune activation (15–17). In chronic infections, prolonged antigen exposure typically culminates in T cell terminal exhaustion with impaired effector functions (18–20). Despite this, adults who exhibit spontaneous control of viremia (elite controllers) maintain viral and immune control for decades by mounting an effective and polyfunctional HIV-specific CTL response characterized by high proliferative capacity (21) and populated with central memory (22, 23) and stem-like memory (24) subsets that support sustained and nonexhausted antiviral activity. Also, PD-1^+^CD8^+^ T cells expressing the transcription factor TCF-1 have been recognized by their memory and proliferative potential as capable of generating effector cells in the context of chronic antigen load in murine (25–28) and human (24, 29) studies. Consequently, the induction of stem-like T cells capable of proliferating and sustaining the burst of cytotoxic effector cell activity required for immune control has been proposed as a strategy for T cell–based immunotherapies in HIV (24).

The low levels of systemic inflammation and immune activation on CD4^+^ T cells seen in PSPs can to some extent be achieved by initiating ART shortly after birth in infants living with HIV after intrauterine transmission, since transmission usually occurs at a late stage of pregnancy (30, 31). Very early ART initiation can also block viral seeding and decrease reservoir size and diversity (31–33). Also, integrated proviral DNA is mainly derived from the transmitted founder virus, which typically has low replicative capacity after mother-to-child transmission (34), and is associated in adults with low reservoir size, low levels of immune activation, and slow disease progression (35). These unique elements together suggest the possibility that very early ART initiation immediately after birth can substantially facilitate the ultimate achievement of functional cure/remission in children living with HIV after in utero infection.

Although remission/posttreatment control has been described anecdotally in a small number of children (36–38), in the great majority of cases, very early ART alone is not sufficient to achieve viremic control after analytical treatment interruption (ATI) (39, 40). Therefore, there is a rationale to explore adjunctive therapies that boost the immune response in early-treated, virally suppressed children at an age when they can mount a more robust Th1 response. Spontaneous control of viremia (elite control) is rare in pediatric infection but, in those cases described, is usually achieved only after 5–10 years of viremia (41, 42). Thus, immune control of viremia in ART-naive children is preceded by a prolonged period of nonprogression despite high viremia. Unlike adult elite controllers, but in common with adults in remission/posttreatment control (43), pediatric elite controllers are not particularly enriched with the well-known protective HLA-B molecules. We previously described in pediatric elite controllers a characteristic T cell compartment with very low immune activation levels and a robust Gag-specific polyfunctional and CTL response (42).

Here, we sought to investigate early T cell markers associated with slow progression in a historical cohort of early-treated infants with HIV followed in 2002–2005 in Durban, South Africa (PEHSS, Paediatric Early HAART and Structured Treatment Interruption Study) (5, 44) who underwent ATI after 12 months of ART. As observed previously in natural hosts of SIV (12), early PD-1 expression on CD8^+^ T cells was the key predictor of slow progression, leading us to explore further the phenotype and function of PD-1–expressing cells in the PSP population.

## Results

### Early PD-1 expression on CD8^+^ T cells before ATI predicts disease progression.

**Figure 1:**
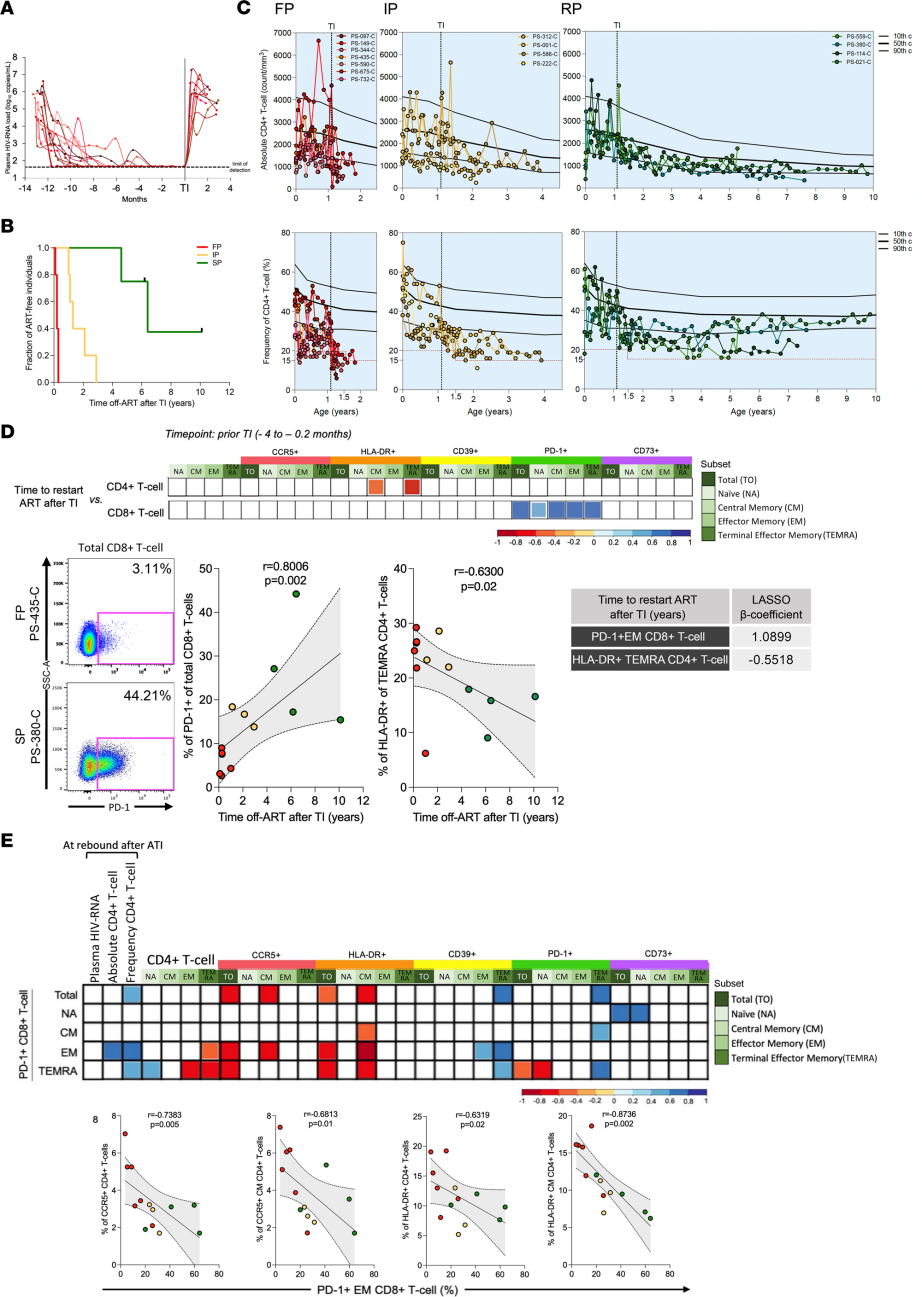
Upregulation of PD-1 on CD8^+^ T cells before ATI is associated with slow progression. (**A**) Longitudinal plasma HIV-RNA for all infants who underwent analytical treatment interruption (ATI) after 1 year on ART. The horizontal dashed line represents the limit of detection and the vertical line the time of ATI. (**B**) Time to restart ART after ATI for each participant according to their group: rapid progressors (RP), intermediate progressors (IP), and slow progressors (SP). FP, fast progressors. (**C**) Individual longitudinal absolute and relative CD4^+^ T cell count in each group. The 10th, 50th, and 90th percentiles for HIV-uninfected children are represented by the 3 black lines. Hashed red line represents the threshold to restart ART according to the 2003 WHO guideline (≤20% if younger than 18 months or 15% if older than 18 months of age). (**D**) Markers before ATI significantly associated with time to restart ART after ATI are shown in the correlation matrix. FACS plot showing PD-1 expression on CD8^+^ T cell in an FP and an SP. HLA-DR on terminal effector CD4^+^ T cells and PD-1^+^ on effector memory CD8^+^ T cells were selected by the LASSO model that best associated with time to restart ART after ATI. (**E**) Markers on CD4^+^ T cells before ATI significantly associated with PD-1 expression on CD8^+^ T cell subsets before ATI. Spearman’s rank tests were used for correlations. The best-fit line and 95% confidence bands are shown. Red, yellow, and green dots represent FP, IP, and SP, respectively.

Thirteen infants with HIV were initiated on ART at a median age of 27 days (IQR, 22–42 days) and underwent ATI after 12 months on ART. ART was restarted after ATI according to the national South African and WHO guidelines prevailing at the time of the study (45): clinical pediatric stage III or advanced stage II; or a CD4^+^ T cell percentage of 20% or less at younger than 18 months of age or less than 15% at older than 18 months of age. All participants were virologically suppressed, and CD4^+^ T cell counts were above 20% prior to ATI (Table 1). Viral rebound occurred within a median of 15 days (IQR, 14–25 days) after treatment interruption in all participants (Figure 1A), but the time to reach the criteria to restart ART varied considerably. The infants were further categorized into rapid progressors if they met treatment criteria within 1 year after ATI, intermediate progressors if they met criteria between 1 and 4 years after ATI, and slow progressors if they met criteria more than 4 years after ATI (Figure 1, B and C).

To identify parameters measured prior to ATI that predict immunological progression during ATI, we analyzed cryopreserved PBMCs collected during viral suppression at the time point immediately before ATI for the expression of activation and exhaustion markers on the T cell compartment. HLA-DR expression on central memory and terminal effector memory CD4^+^ T cells negatively correlated with time to restart therapy while unexpectedly, PD-1 expression on all CD8^+^ T cell subsets was positively correlated with slow progression (Figure 1D and Supplemental Figure 1; supplemental material available online with this article; https://doi.org/10.1172/jci.insight.156049DS1). In the Least Absolute Shrinkage and Selection Operator (LASSO) model, where the duration of viral suppression during the first year, age at ART initiation, and CD4^+^ T cell counts were also included, PD-1 expression on effector memory CD8^+^ T cells was the primary variable selected and associated with slow progression.

Higher PD-1 expression, especially on effector memory CD8^+^ T cells, was associated with lower CCR5^+^ and HLA-DR^+^ on total and central memory CD4^+^ T cells before ATI, two cardinal features previously identified in PSPs (Figure 1E) (11). High PD-1 expression on effector memory CD8^+^ T cells was also associated with lower PD-1 expression on total CD4^+^ T cells, with a higher CD4/CD8 ratio and with higher absolute CD4^+^ T cell count immediately after the viral rebound.

To characterize the CD8^+^ T cell population expressing PD-1, we looked at the coexpression of activation and exhaustion markers. PD-1^+^CD8^+^ T cells in the rapid progressors expressed more CD39 when compared with slow progressors (Supplemental Figure 2). PD-1 when coexpressed with CD39, an E-NTPDase classically upregulated in activated cells, characterizes a CD8^+^ T cell subset with impaired effector function and increased expression of other inhibitory receptors (17). To further analyze the PD-1^+^CD8^+^ T cells, we compared the genes expressed in this population in rapid versus slow progressors. We undertook transcriptomic analysis of populations sorted by PD-1 expression on CD8^+^ T cells in 3 rapid progressors and 3 slow progressors (sample availability limited the numbers that could be studied). Although the transcriptomic analysis in sorted PD-1^+^CD8^+^ T cells from the time point immediately before ATI showed that a large proportion of differentially expressed genes appeared to be driven by individual heterogeneity (Supplemental Figure 3A), the rapid progressors showed higher levels of the inhibitory receptor Tim-3 (*HAVCR2*), a marker when coexpressed with PD-1^+^ that also characterizes terminally exhausted CD8^+^ T cells (Supplemental Figure 3B), while slow progressors showed upregulation of pathways directly related to antiviral function, cell proliferation, and sustained effector activity (Supplemental Figure 3C).

### Early upregulation of PD-1 on CD8^+^ T cells predicts stronger HIV-specific responses in infants after ATI.

We hypothesized that early upregulation of PD-1 would act as “damage control” and limit the hyperactivation and terminal exhaustion of the CD8^+^ T cell compartment that is commonly seen with disease progression (15–17) while preserving the effector potential of this population.

To investigate whether PD-1 expression on CD8^+^ T cells prior to ATI led to stronger and preserved antiviral activity after viral rebound, we used ELISPOT assays to examine the HIV-specific responses in individuals at 3 months after initiation of the ATI who had not restarted ART. Viral loads at the time point selected for ELISPOT analysis were high and similar in the 3 groups (Supplemental Table 1). This time point was chosen because beyond this time the rapid progressor group had met the criteria to restart ART. It is worth noting that the median viral load at diagnosis in these infants was 721,000 HIV RNA copies/mL, and ART was initiated at a median of 26 days, at which time the median viral load was 851,000 HIV RNA copies/mL. The median time to suppression of viremia on ART was 4.3 months (Figure 1A). Thus, these infants certainly had sufficient antigen exposure to stimulate PD-1 expression on CD8^+^ T cells.

**Figure 2:**
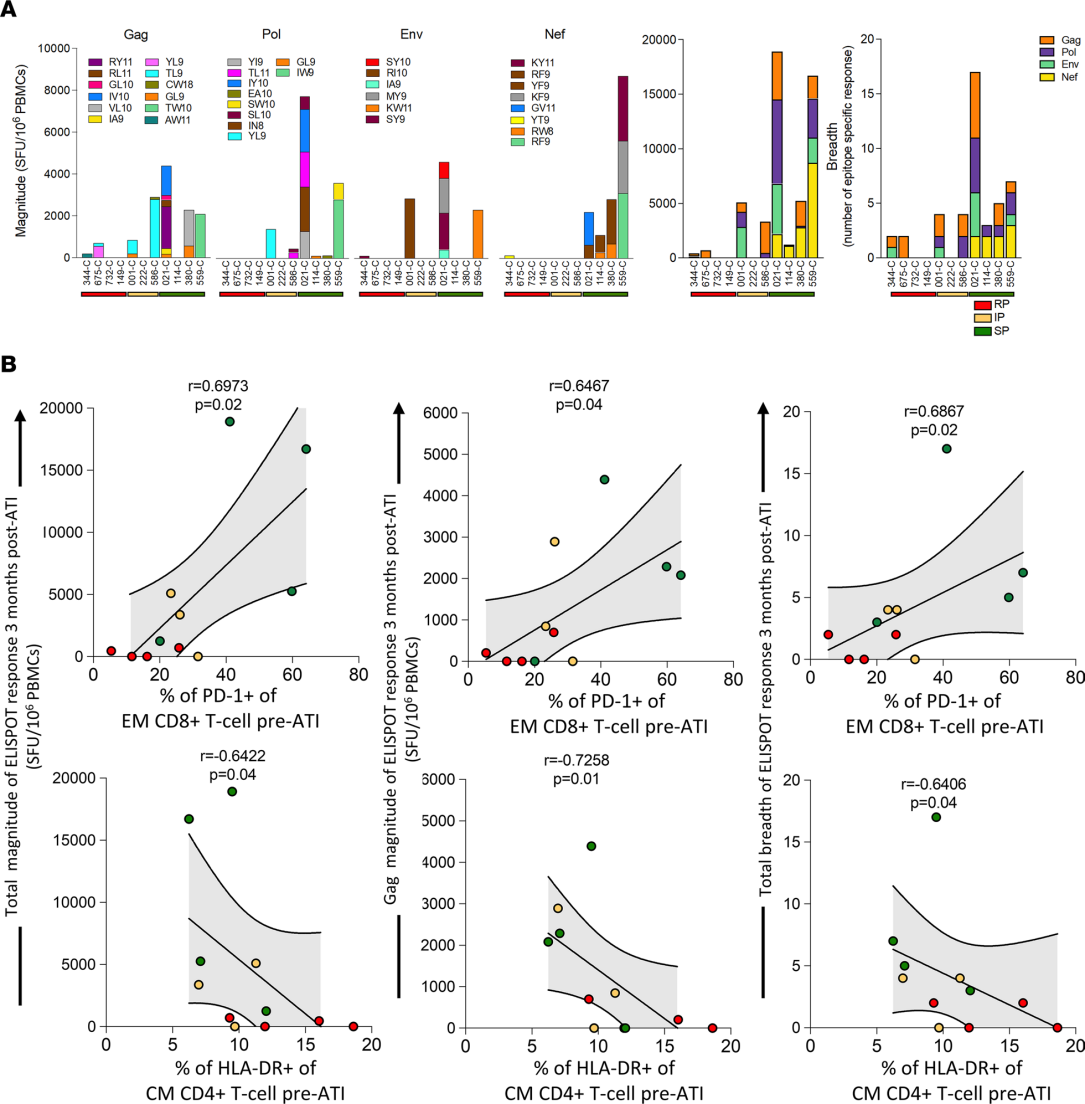
PD-1 expression on CD8^+^ T cells before ATI is associated with higher HIV-specific CD8^+^ T cell response after ATI in the cohort of early-treated infants. (**A**) Gag, Pol, Env, Nef, and total magnitude and total breadth ELISPOT response in 4 RP (red), 3 IP (yellow), and 4 SP (green) 3 months after ATI. Epitope colors were selected to better differentiate their contribution in the bar chart. (**B**) Total and Gag magnitude and total breadth of ELISPOT response correlated with PD-1^+^ effector memory CD8^+^ T cells and HLA-DR^+^ central memory CD4^+^ T cells before ATI. Spearman’s rank tests were used for correlations. The best-fit line and 95% confidence bands are shown. Red, yellow, and green dots represent RP, IP, and SP, respectively.

Slow progressors tended to have higher magnitude and breadth of HIV-specific CD8^+^ T cell response when compared with rapid progressors, although with only a small number in each group, this was not statistically significant (Figure 2A). However, the total magnitude of HIV-specific CD8^+^ T cell responses (Gag, Pol, Env, and Nef) 3 months after ATI positively correlated with pre-ATI PD-1 expression on CD8^+^ T cells (*P* = 0.02) and negatively with pre-ATI HLA-DR^+^ expression on CD4^+^ T cells (*P* = 0.04) (Figure 2B). Similar findings were observed for the Gag-specific response alone and for breadth of the total HIV-specific response. These data are consistent with the notion that in these infants, PD-1 expression on effector memory CD8^+^ T cells prior to ATI preserves the CD8^+^ T cell compartment and predicts a stronger antiviral response during ATI.

### CD8^+^ T cells from PSPs have a stem-like PD-1^+^ phenotype.

In adults infected with HIV, PD-1 expression is typically associated with T cell exhaustion and impaired effector function and is a signal of HIV disease progression (18, 46). When we evaluated the expression of PD-1 on CD8^+^ T cell populations in a group of 182 ART-naive children, PD-1 was only positively associated with immune activation in children older than 5 years but not in the younger group (Supplemental Figure 4), suggesting a different role of PD-1 or different populations of PD-1–expressing T cells in early life compared with later childhood and adolescence.

To investigate how PD-1 expression and associated markers on T cells alter throughout childhood, we further studied 2 of the 4 slow progressors from the ATI study across 7–10 years of follow-up. Neither of them met the prevailing WHO criteria to restart ART throughout this time, and they had similarly stable plasma HIV-RNA load (median, 73,806, and 85,892 copies/mL); however, their CD4^+^ T cell trajectories differed substantially (Supplemental Figure 5). Both these slow progressors met the criteria for PSPs in that they maintained absolute CD4^+^ T cell counts of more than 350 cells/mm^3^ in the absence of ART at age 5 years or above. In study participant PS-021-C, whose relative CD4^+^ T cell count increased from a nadir of 20% to a peak of 40% at 9.8 years, PD-1 expression on CD8^+^ T cells remained stable at a moderate level in association with high expression of CD73, a marker associated with memory maintenance (47, 48). By contrast, study participant PS-114-C, who was close to the criteria for restarting ART throughout the follow-up period, experienced an upregulation of PD-1 and HLA-DR and downregulation of CD73 on CD8^+^ T cells.

**Figure 3:**
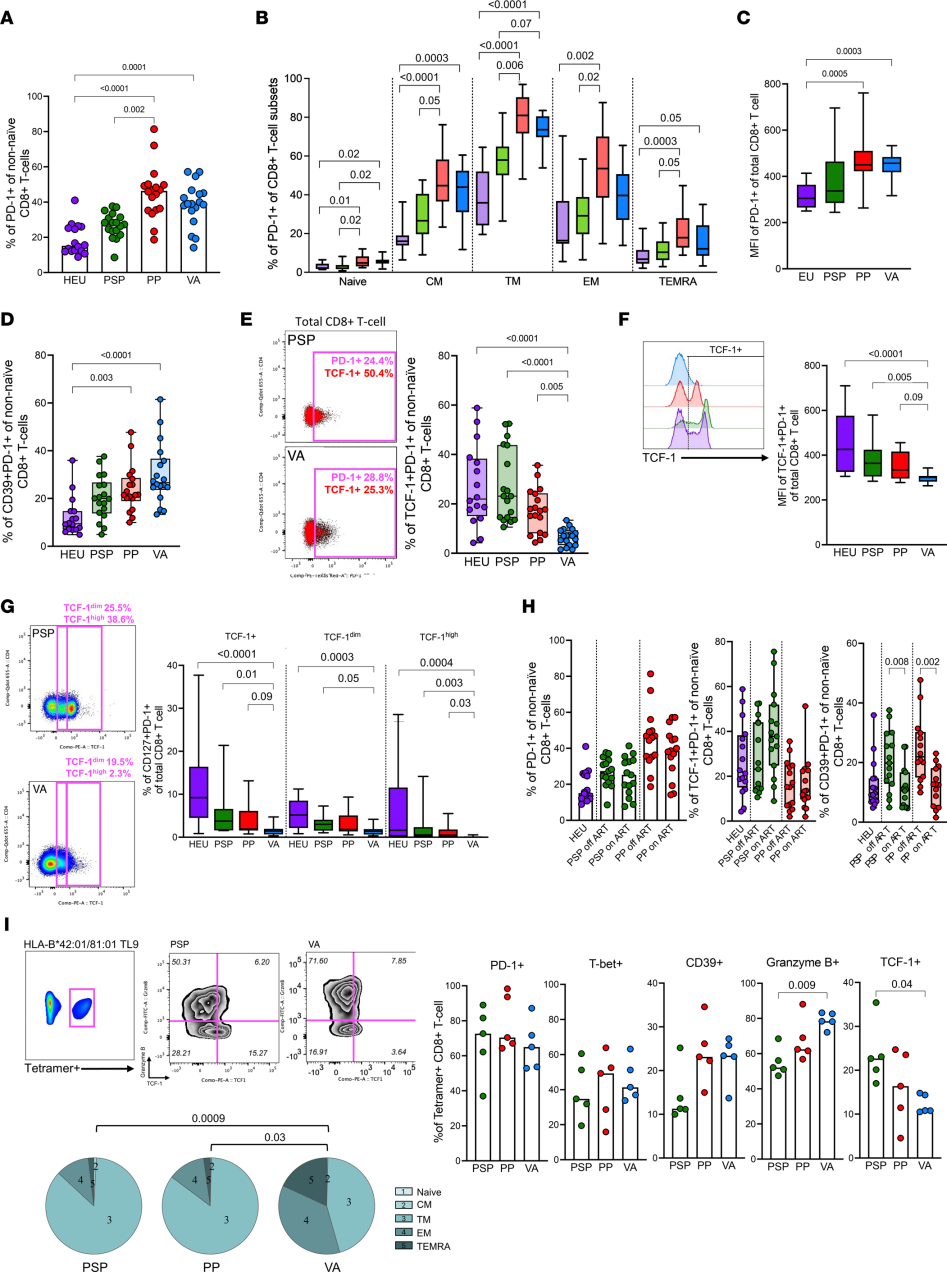
PD-1 expression on nonnaive CD8^+^ T cells is characterized by a stem-like TCF-1^+^ phenotype in ART-naive pediatric slow progressors. (**A–C**) Frequency of PD-1^+^ on nonnaive CD8^+^ T cells (**A**) and on memory (**B**) CD8^+^ T cells and absolute levels of PD-1 of total CD8^+^ T cells (**C**) measured by MFI among HIV-exposed uninfected children, PSPs, pediatric progressors, and viremic adults. (**D**) Frequency of CD39^+^PD-1^+^ of nonnaive CD8^+^ T cells as a marker of terminal exhaustion. (**E**) Typical FACS plot showing PD-1^+^ population (pink gate) and TCF-1^+^ population (red dots) in PSPs and viremic adults. Frequency of TCF-1^+^PD-1^+^ of nonnaive CD8^+^ T cells. (**F**) Typical histogram and MFI levels TCF-1^+^ on PD-1^+^ of total CD8^+^ T cells. (**G**) FACS plot showing typical frequency of TCF-1^+^ of CD8^+^ T cells in PSPs and viremic adults. Frequency of CD127^+^PD-1^+^ of total CD8^+^ T cells according to TCF-1 expression levels (TCF-1^hi^, TCF-1^dim^, and TCF-1^neg^). (**H**) Pairwise comparison of frequency of PD-1^+^, CD39^+^PD-1^+^, and TCF-1^+^PD-1^+^ of nonnaive CD8^+^ T cells before and after 1–2 years of ART initiation and viral suppression. (**I**) Typical FACS plot of Tetramer^+^ (Gag-TL9)-specific CD8^+^ T cells showing Granzyme B and TCF-1 gating in a typical PSP and viremic adults. Frequency of PD-1^+^, T-bet^+^, CD39^+^, Granzyme B^+^, and TCF-1^+^ on Tetramer^+^ CD8^+^ T cells for each group. Memory subset distribution on Tetramer^+^ CD8^+^ T cells. Statistical comparison between 3 and 4 groups was done using a Kruskal-Wallis test followed by Dunn’s test to correct for multiple comparisons. Wilcoxon matched-pairs test was performed for pairwise comparisons.

Next, we investigated the phenotype of PD-1–expressing CD8^+^ T cells in a group of older ART-naive PSPs (*n* = 18; median, 12.1 years; IQR, 10.9–15.2) and pediatric progressors (*n* = 18; median, 13.4 years; IQR, 11.9–16.8), matched by age and sex with a group of HIV-exposed uninfected children (*n* = 16; median, 13.8 years; IQR, 8.3–16.9). Of note, none of the slow progressors described above were included in the older PSP group. A fourth group of horizontally infected ART-naive viremic adults (*n* = 18; median, 29.0 years; IQR, 26.0–35.5) was included for comparison (Supplemental Table 2). All pediatric and adult participants were off ART. As reported in previous studies, the frequency of PD-1^+^CD8^+^ T cells (excluding the naive subset) was higher in the HIV-infected groups (Figure 3A) and at greater levels in transitional memory and effector memory subsets (Figure 3B). PD-1–expressing nonnaive CD8^+^ T cells were significantly lower in frequency in PSPs when compared with pediatric progressors (*P* = 0.002). The absolute levels of PD-1 expression measured by MFI were also higher in pediatric progressors and viremic adults when compared with HIV-exposed uninfected children (Figure 3C). Similar to the findings observed in the participants undergoing ATI, terminally exhausted CD39^+^PD-1^+^ nonnaive CD8^+^ T cells were increased in pediatric progressors (*P* = 0.003) and viremic adults (*P* < 0.0001) but not in PSPs when compared with HIV-exposed uninfected children (Figure 3D). When associated with TCF-1, PD-1–expressing T cells are characterized by a stem-like phenotype with increased proliferative capacity (27). We found a higher frequency of TCF-1^+^PD-1^+^ on nonnaive CD8^+^ T cells in the pediatric groups (*P* < 0.0001) when viremic adults were compared with HIV-exposed uninfected children or with PSPs (Figure 3E). The small TCF-1^+^ population in viremic adults also showed the lowest MFI levels (Figure 3F), and a TCF-1^hi^ population was only apparent in PSPs and HIV-exposed uninfected children (Figure 3G). Moreover, PD-1^+^TCF-1^+^ was highly associated with the expression of CD127, the IL7Rα required in the generation of long-term memory cells. Although TCF-1^+^PD-1^+^CD8^+^ T cells have been associated with the expression of CXCR5 in murine models, we did not observe this correlation in the pediatric group.

Some participants in the previous analysis started ART because of clinical progression or change in national guidelines to universal treatment. Consistent with the previous findings and with adult data (18, 49), the terminally exhausted CD39^+^PD-1^+^ population shrank after 1–2 years of ART initiation and viral suppression in PSPs and pediatric progressors (*P* = 0.008 and *P* = 0.002, respectively), while the long-lived TCF-1^+^ population relatively expanded (Figure 3H).

To determine whether the findings observed in bulk CD8^+^ T cells were also present in HIV-specific CD8^+^ T cells, we generated HLA-B*42:01–restricted and HLA-B*81:01–restricted TL9-Gag–specific tetramers to characterize HIV-specific CD8^+^ T cells in selected individuals in PSPs, pediatric progressors, and viremic adults (Figure 3I). We observed similar frequencies of PD-1^+^ and T-bet^+^ on HIV-specific cells in the 3 groups. However, Granzyme B was significantly higher, and TCF-1 expression was lower in viremic adults than PSPs (*P* = 0.009 and *P* = 0.04, respectively) on HIV-specific CD8^+^ T cells. There was also a tendency for higher levels of CD39^+^ on antigen-specific CD8^+^ T cells in the viremic adults. As expected, the CD8^+^ T cell compartment in viremic adults was markedly populated with more differentiated subsets than in PSPs and pediatric progressors (*P* = 0.009 and *P* = 0.03, respectively). These findings indicate an HIV-specific CTL population enriched with a stem-like phenotype in the PSP group. To test whether the constant TCR stimulation in viremic individuals affects the PD-1^+^TCF-1^+^ population, we stained cells from the same individuals with tetramers to identify CMV-pp65–specific CD8^+^ T cells. The frequency of PD-1^+^, CD39^+^, and TCF-1^+^ populations was lower in the CMV-specific CD8^+^ T cells than in the HIV-specific CD8^+^ T cells, and no differences were observed among the 3 groups (Supplemental Figure 6), highlighting the importance of antigen load for the survival of these PD-1^+^ antigen-experienced CTLs.

### HIV-specific CD8^+^ T cells have stronger proliferative potential in PSPs.

**Figure 4:**
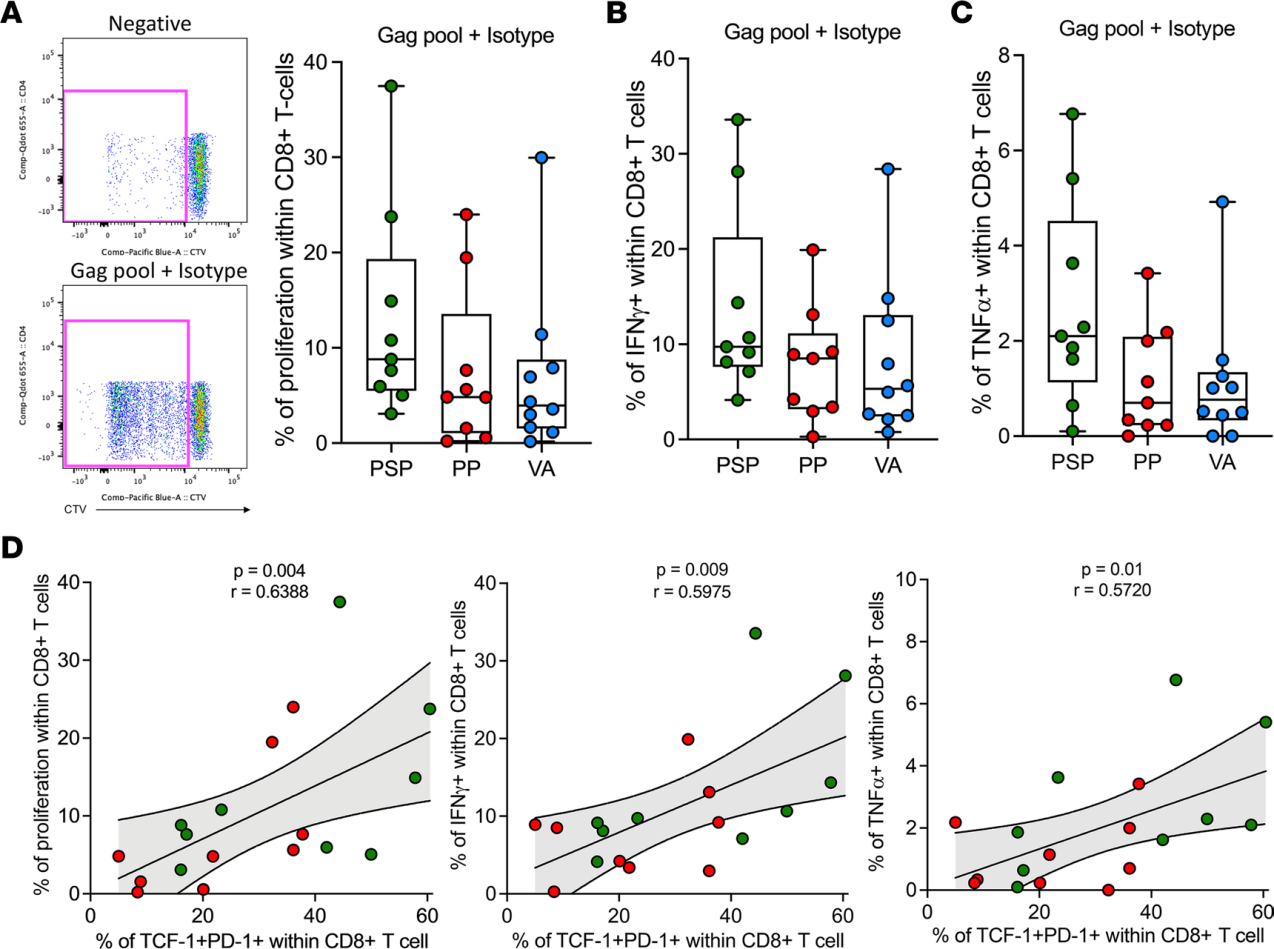
Stem-like PD-1^+^CD8^+^ T cells positively correlate with HIV-specific response. (**A**) Typical FACS plot of the proliferation rate of unstimulated and Gag-stimulated CellTrace Violet–labeled CD8^+^ T cells. Frequency of proliferated CD8^+^ T cells after 7 days of stimulation with Gag-pool. (**B** and **C**) IFN-γ^+^ (**B**) and TNF-α^+^ (**C**) on proliferated CD8^+^ T cells. (**D**) Frequency of total, IFN-γ^+^, and TNF-α^+^ proliferated CD8^+^ T cells correlated with frequency of TCF-1^+^PD-1^+^ of total CD8^+^ T cells. Green and red dots represent PSPs and pediatric progressors, respectively.

To investigate whether the higher frequency of the antiviral-specific TCF-1^+^PD-1^+^CD8^+^ T cell population in PSPs translates to higher proliferative capacity, CellTrace Violet–labeled PBMCs were stimulated for 7 days with HIV-Gag or CMV-pp65 pools. Although not statistically significant, we observed a tendency to a higher frequency of proliferating CD8^+^ T cells in PSPs than in pediatric progressors and viremic adults in PBMCs stimulated with Gag (Figure 4A). In the final 12 hours of incubation, we boosted the stimulation with the same Gag and pp65 peptide pools to measure the ability of these proliferated CD8^+^ T cells to secrete cytokines. Again, we observed a tendency toward higher IFN-γ^+^ and TNF-α^+^ proliferated CD8^+^ T cells in PSPs (Figure 4, B and C). The frequency of TCF1^+^PD-1^+^CD8^+^ T cells prior to stimulation trended toward a positive correlation with the frequency of total proliferating CD8^+^ T cells and with IFN-γ^+^CD8^+^ T cells and TNF-α^+^CD8^+^ T cells (Figure 4D). As anticipated by the immunophenotype, when we looked at the population stimulated with the pp65-CMV pool, we observed an overall lower proliferation rate and fewer IFN-γ^+^CD8^+^ T cells and TNF-α^+^CD8^+^ T cells compared with HIV-Gag–stimulated PBMCs, with no difference between the 3 groups (Supplemental Figure 7).

## Discussion

To increase the number of individuals in whom posttreatment control can be achieved, it is important to identify mechanisms that promote potent and sustained antiviral responses and facilitate immediate viral control after ART cessation. Within a group of early-treated infants who underwent ATI after 1 year on ART, we showed that early expression of PD-1 on CD8^+^ T cells strongly predicted slower HIV disease progression during subsequent ATI and was strongly associated with the cardinal features observed previously in ART-naive pediatric long-term nonprogressors: low HLA-DR expression and low CCR5 expression on the long-lived central memory CD4 T cell subset (11, 50). This PD-1^+^CD8^+^ T cell population, representing a nonterminally exhausted phenotype with preserved effector potential, predicts a stronger HIV-specific CTL response during ATI. When we investigated PD-1^+^CD8^+^ T cells in ART-naive adolescent PSPs, we identified an enrichment of the TCF-1^+^ population that was associated with higher proliferative capacity and stronger Gag-specific effector functionality. In contrast, ART-naive progressors and viremic adults were enriched with a more terminally exhausted subset of PD-1^+^CD8^+^ T cells. Although it is challenging to address the cause-and-effect relation of viral control and T cell immune activation/exhaustion in a cross-sectional analysis, we cannot exclude the impact of differences in plasma viral load and the results observed here (Supplemental Table 1).

PSPs, adult viremic nonprogressors, and natural hosts of SIV appear to share the same fundamental immunological adaptations that contribute to maintaining normal CD4^+^ T cell counts despite ongoing viral replication, which result in low immunodeficiency virus infection of the long-lived CD4^+^ T cell populations (6, 10, 11, 51). In experimental studies in nonhuman primates, both pathogenic (Rhesus monkeys) and nonpathogenic (African green monkeys) models upregulate genes associated with immune activation in CD4^+^ T cells during acute SIV infection (13). However, this is followed by a natural downregulation of these genes in nonpathogenic SIV, leading to the tolerogenic state of low immune activation and viral replication in the chronic phase. Rapid upregulation of PD-1 on CD8^+^ T cells located in the lymph nodes has been temporally associated with resolution of immune activation in sooty mangabeys and proposed as an important mechanism to control overactivation of the cytotoxic response and avoid tissue damage. In the pathogenic SIV infection model, this early upregulation of PD-1 is not seen, and PD-1 is upregulated during the progression phase (12). Similarly, in the ART-treated infants who underwent subsequent ATI described here, PD-1 expression on CD8^+^ T cells predicted slower HIV disease progression and negatively correlated with immune activation and CCR5 expression on the CD4^+^ T cells. PD-1 was only positively associated with higher immune activation in older ART-naive children. Interestingly, in a recent anecdotal case of remission/posttreatment control in a vertically infected child, the PD-1 level, but not HLA-DR and CCR5, was elevated on CD4^+^ and CD8^+^ T cells (38).

The PD-1^+^CD8^+^ T cells in the slow progressor group evaluated prior to ATI did not express the classical terminal exhausted phenotype at the transcriptional level but demonstrated lower Tim-3 gene expression and the upregulation of pathways associated with sustained effector functions when compared with rapid progressors. PD-1 expression on CD8^+^ T cells prior to ATI also correlated with the magnitude of virus-specific CD8^+^ T cell responses 3 months after ATI. However, as has been well-documented, in the first 2 years of life, these HIV-specific CD8^+^ T cell responses have a much more modest antiviral effect compared with those generated in later childhood and adulthood (52, 53), for reasons related to the more tolerogenic and Th2 polarized immune response in young children (2, 3, 54). Nonetheless, the observed phenotype contributes to avoiding hyperstimulation of the CD8^+^ T cell compartment and to preserving CD4 T cell numbers and function (11) as well as antigen-specific CD8^+^ T cells that are not terminally exhausted and ready to be exploited as the child matures in age.

Immune responses alter from early life through childhood and adolescence into adulthood (55). Children who are infected with HIV and yet maintain normal-for-age CD4^+^ T cell counts with low levels of immune activation despite persistent high viremia are relatively common, around 10% at 5 years old, reducing to approximately 5% at 10 years old (11), whereas the equivalent adult viremic nonprogressors are exceptionally rare (<0.01%, or even <0.001%) (6–9). It is clearly the case that the early life immune response is more tolerogenic and tightly regulated than the more aggressive immunity seen in adults and in broad terms, this likely underlies the observation of a high frequency of viremic nonprogressors in early life and a low frequency in adult life. The discordant relationship between PD-1 expression and CD4^+^ T cell activation in children younger and older than 5 years, respectively, serve to highlight the fact that first, immune responses alter by age toward a more adult-like response, and second, and more specifically, PD-1 expression is not necessarily the consequence of chronic immune activation and T cell exhaustion. The studies here of the older children/adolescents, which still include a group of PSPs, showed that, although the majority have transitioned more toward adult-type (versus early childhood-type) responses, there remains a minority of children/adolescents represented by the PSPs in whom the early childhood type of responses have persisted, with higher frequencies of the stem-like phenotype of PD-1–expressing CD8^+^ T cells observed.

The HIV-specific CD8^+^ T cell population expressing PD-1 and TCF-1 represents a promising target for T cell therapies because of the long-term survival and proliferative capacity of these stem-like cells. Superior viremic control in adult elite controllers is associated both with a stronger proliferative capacity of HIV-specific CTLs (21, 24) and with the presence of long-lived memory populations (22, 23), including the TCF-1^+^ stem-like memory subset (24). Reinforcing their recall ability in chronic infections, TCF-1^+^PD-1^+^CD8^+^ T cells led to the expansion of virus-specific CTLs upon stimulation even after months of antigen cessation in patients with hepatitis C virus infection (29). However, expanded TCF-1^+^ cells need to downregulate TCF-1 to allow themselves further differentiation into effector CTLs. Although we did not sort TCF-1^+^ cells to observe its downregulation in proliferated cells, we observed a positive correlation between the proportion of TCF-1^+^CD8^+^ T cells prior to stimulation and the frequency of antiviral function by the IFN-γ expression in proliferated Gag-specific CTLs after 7 days of stimulation.

In addition to the acquisition of effector functions, TCF-1^+^ CTLs also need to migrate to lymphoid tissues to target the viral reservoir. Interestingly, in mouse models, virus-specific stem-like TCF-1^+^PD-1^+^CD8^+^ T cells have been associated with CXCR5 expression (26). Although our data and a previous study did not find such correlation in peripheral blood in humans, PD-1^+^CXCR5^+^CD8^+^ T cell frequency in peripheral blood was recently associated with better effector function and higher CD4^+^ T cell counts in adult HIV infection (56).

The initiation of ART shortly after birth following in utero infection decreases the period of viremia and seeding of the viral reservoir, protecting the T cell compartment from chronic antigen stimulation (31). In adults, immune activation and exhaustion can only be partially reversed with ART even when started during primary infection (57). By contrast, the T cells in early-treated infants can be more effectively preserved by long-term viral suppression (31) until children can generate a more potent Th1 polarized effector response. The presence of a preserved T cell compartment that is not terminally exhausted can be explored in immunotherapeutic approaches that generate and boost HIV-specific responses in older children. Although rare, children controlling viral replication exist (41). Pediatric elite controllers only achieve viremic control at an older stage by maintaining a very low level of immune activation and exhaustion while mounting a Gag-specific response that is highly polyfunctional (42), usually in the absence of the HLA-B alleles that are associated with elite control in adult infection (58, 59).

It is important to highlight the limitations of this study. Although PSPs show similarities to natural hosts of SIV infection in maintaining normal-for-age CD4 counts and low levels of immune activation despite high viremia (11), the differences in immune activation and exhaustion in the PSP and pediatric progressor analysis of older children/adolescents can be partially explained by the differences in plasma viremia between the two groups. In addition, most analyses were undertaken in bulk CD8^+^ T cells, which limits our capacity to interpret the results with respect to HIV-specific CD8^+^ T cells. Sample availability from this historical cohort prevented transcriptomic evaluations in more than 3 individuals in each group. Also, in this analysis, PD-1^hi^ cells were sorted, which decreased our capacity to capture differences between the slow and rapid progressor groups, since PD-1 is expressed at a moderate level in the stem-like CD8^+^ T cell subset. Limited sample availability from the historical PEHSS cohort also prevented further analyses of the function and phenotype of the PD-1^+^ cells that arise early in infection and after ATI. Epigenetics play an essential role in modulating PD-1 gene expression and can be a critical factor in identifying mechanisms leading to early PD-1 upregulation and HIV slow progression. Finally, it would be optimal to explore the tissue-resident cells and their role in HIV pathogenesis instead of extrapolating findings obtained with peripheral blood cells, which was not possible because of the logistical and ethical challenges in sampling lymphoid tissue in infants. However, studies of the CXCR5^+^ population of TCF-1^+^CD8^+^ T cells in murine models (26) support the need to evaluate human lymphoid tissue to assess the immense potential of stem-like CD8^+^ T cells in future T cell therapeutic strategies for HIV cure/remission.

The findings here make a potentially novel contribution to understanding mechanisms contributing to slower disease progression in children and add to the previously described similarities with the mechanisms underlying nonprogression in natural hosts of SIV (11, 50). Moreover, the phenotype of stem-like CD8^+^ T cells that is associated with HIV-specific CTLs with high proliferative capacity can be explored in future adjuvant therapies in the pursuit of HIV remission. Despite the limited analyses of function and phenotype of the HIV-specific CD8^+^ T cell population in this study, the data presented here also strengthen the notion that, owing to the immune differences that exist in early life compared with adulthood, remission may be more easily achievable in children. In future work, it will be important to identify therapeutic candidates that can harness the high proliferative capacity and long-term survival of PD-1^+^TCF-1^+^ virus-specific CTLs while at the same time exploiting their cytotoxic potential in individuals living with HIV.

## Methods

### Study population.

The PEHSS cohort was a feasibility study to investigate different approaches of starting ART in infants infected with HIV (5, 44) that was undertaken in 2002–2005, prior to implementation of universal ART in all infants infected with HIV. We evaluated participants randomized to arm B, who received immediate ART from birth to 12 months followed by ATI. ART was restarted after ATI according to the prevailing 2003 WHO and South African national guidelines. To investigate T cell markers associated with disease progression or time to meeting ART criteria, we used PBMCs from 13 participants who were virally suppressed from whom samples were available up to 3 months before ATI. Multiple samples from 2 study participants, PS-021-C and PS-114-C, were obtained for the longitudinal study.

In addition, we investigated the immunophenotype of PD-1–expressing CD8^+^ T cells in older children and young adults from previously described cohorts in sub-Saharan Africa. PSPs were defined as children who were infected with HIV, ART-naive, and older than 5 years with CD4^+^ T cell counts of more 350 cells/mm^3^ and more than 20%; pediatric progressors were defined as having CD4^+^ T cell counts of less than 350 cells/mm^3^ or less than 20%. Viremic adults (viremic adults) consisted of horizontally infected ART-naive individuals with chronic infection matched by sex. The fourth group consisted of HIV-exposed uninfected children matched by age and sex. No data were available on viral load and drug suppression for the mothers of these children. In analyses done after ART initiation, samples were selected from the time point after 1 to 2 years of viral suppression.

### Immunophenotype of cryopreserved PBMCs.

Cryopreserved PBMCs were thawed and cells rested in R10 medium for 3 hours at 37°C in 5% CO_2_. Then, cells were washed with PBS and stained with Live/Dead near-IR stain (Invitrogen) according to the manufacturer’s instructions. After incubation, cells were washed again and stained for 30 minutes at 4°C in FACS buffer containing the surface antibody mix. PBMCs were fixed and permeabilized with Cytofix/Cytoperm solution (BD Biosciences) for 45 minutes at 4°C and stained with the intracellular antibody mix) for 30 minutes at 4°C. Cells were washed and acquired on a BD Biosciences LSR II. The complete fluorochrome-conjugated antibody list is in Supplemental Table 3.

### Tetramer and staining of virus-specific CD8^+^ T cells.

Four sets of peptide-HLA tetramers conjugated with BV421 were obtained from ImmunAware ApS: HLA-B*42:01/Gag-TL9 (TPQDLNTML), HLA-B*42:01/pp65-RV10 (RPHERNGFTV), HLA-B*81:01/Gag-TL9 (TPQDLNTML), and HLA-B*81:01/pp65-GL8 (GPISGHVL). Two million cryopreserved PBMCs were thawed and stained according to the manufacturer’s instructions. The subsequent staining protocol followed the description above. Samples were acquired on a BD Biosciences LSR II.

### Cell sorting, RNA library preparation, and RNA-Seq.

We selected 3 slow progressors and 3 rapid progressors with cryopreserved PBMCs available at the time point before ATI. After thawing and staining, 100 PD-1^+^CD8^+^ T cells were sorted in triplicates using a FACSAria Fusion (BD Biosciences) directly into RLT buffer (Qiagen) containing 1% β-mercaptoethanol and stored at –80°C. RNA extraction, cDNA conversion, and whole-transcriptome-amplification were done using Smart-seq2 as previously described (60). Quality and concentration of the material amplified were confirmed with BioAnalyzer (Agilent Technologies) and Qubit assay kit (Thermo Fisher Scientific). The process was followed by tagmentation and amplification of diluted samples and barcoding using Nextera XT DNA Library prep kit (Illumina). Samples were cleaned using AMPure XP SPRI beads (Beckman Coulter) and sequenced on a HiSeq system using HiSeq SBS Kit v4 (Illumina) to an average depth of 10 × 10^6^ reads.

The sequenced data were analyzed as follows: the sequences were demultiplexed using bcl2fastq snapshot 5 (mask_short_adapter_reads 22, minimum_trimmed_read_length 35) before alignment. FASTQs were aligned using the smartseq2 snapshot 8 workflows using “GRCh38_ens93filt” as the reference genome. Both protocols were adopted from the Cumulus workflow. The cell by gene matrix was subsequently loaded and analyzed using DESeq2 (v 1.2.8.1) in R. Samples with fewer than 9 million total reads and genes not found in more than 3 samples, or greater than a sum of 5 counts were excluded from further analysis. Differential gene expression was conducted using the Wald test. Gene set enrichment analysis (GSEA) was performed on genes pre-ranked based on the Wald statistic and analyzed via GSEA version 4.0.3 using the curated Hallmark gene set. Significant gene sets were filtered based on an adjusted *P* value less than 0.05. The data were deposited in the NCBI’s Gene Expression Omnibus database (GEO GSE221366).

### ELISPOT assays.

ELISPOT assays were performed contemporary to the PEHSS study in freshly isolated PBMCs and previously described and published (52). A confirmatory assay followed a first assay screening for T cell responses. The peptide panel consisted of 18-mers overlapping peptides spanning the entire HIV-1 clade C consensus sequence and used in a matrix system in screening assays. Briefly, peptides were added at a final concentration of 2 μg/mL to a 96-well plate coated with anti-human IFN-γ monoclonal antibody (Mabtech) with 100 μL of R10 medium at 50,000 or 100,000 cells/well. Negative controls contained media only, and positive controls contained phytohemagglutinin. After overnight incubation, IFN-γ–producing cells were revealed after subsequent washes and incubations with IFN-γ monoclonal antibody biotinylated secondary antibody (Mabtech), streptavidin-alkaline phosphatase conjugate antibody (Mabtech), and alkaline phosphatase substrate reagents (Bio-Rad). The number of spots per well was quantified using an automated ELISPOT plate reader (AID ELISPOT reader system; Autoimmun Diagnostika GmbH). A response was defined as positive if there were 100 or more spot-forming cells/million PBMCs and it was 3 or more standard deviations above the negative control after background subtraction. The optimal epitope peptides used were those restricted by the HLA class I molecules expressed by the study participant of interest and listed in the “best-defined HIV-specific CD8^+^ T cell epitope peptide list” compiled by Brander et al. in the Los Alamos database (https://www.hiv.lanl.gov).

### Proliferation assay.

Cryopreserved PBMCs were thawed and rested overnight in R10 at 37°C in 5% CO_2_. Cells were washed with PBS and stained with CellTrace Violet (Life Technologies) for 10 minutes at room temperature. Cold FBS was used to quench the reaction, and labeled PBMCs were resuspended in R10 containing 10% human AB serum (Sigma-Aldrich). Next, 250,000 cells were plated in duplicate in 96-well round-bottom plates in the presence of anti-CD28/anti-CD49d at 1 μg/mL (BD Biosciences), HIV-Gag clade C, or CMV-pp65 pools of overlapping consensus peptides (NIH AIDS Reagent Program) at a final concentration of 2 μg/mL for each peptide. The negative control included DMSO in the same concentration of peptide pool solution and 2 μg/mL phytohemagglutinin L (Sigma-Aldrich) used as a positive control. Cells were incubated for 7 days at 37°C in 5% CO_2_ with a 20% top-up of media on day 4. Twelve hours before the end of incubation, the stimulation was boosted with the same concentration of the respective stimulus, and Brefeldin A at 5 μg/mL (BioLegend) and Monensin at 1 μg/mL (BioLegend) were added to allow the measurement of IFN-γ^+^ and TNF-α^+^ cells. Surface and intracellular staining followed as described.

### FACS analyses.

The data were analyzed in FlowJo v10.6.2 (Tree Star LLC). Positive gates were selected using fluorescence minus one (FMO) and memory subsets were obtained using Boolean Gates. For proliferation and intracellular cytokine staining assays, positive responses were considered after subtracting the background if at least 3 times higher.

### Statistics.

Graphs were created and statistical analysis was done with GraphPad Prism v9.0.1 unless otherwise stated. Continuous variables are shown in median and IQR. Statistical comparison between 3 and 4 groups was done using a Kruskal-Wallis test followed by Dunn’s test to correct for multiple comparisons. A Wilcoxon matched-pairs test was performed for pairwise comparisons. Spearman’s test was used to show correlation and a simple linear regression model for best-fit line and 95% confidence interval. Memory subset distributions and pie charts were done with the permutation test performed by Spice version 6.0. The correlation matrix was built with the *corrplot* package. When multiple covariates were identified in the correlation matrix, the LASSO model was built. LASSO (61, 62) is a penalized multivariate linear regression model to select the relevant predicting covariates. The package *glmnet* was used to obtain the optimal penalty via 10-fold cross-validation. For this study, a *P* value less than 0.05 was considered significant.

### Study approval.

Written informed consent was obtained from all participants prior to participation in the study, except those who were underage, where written informed consent was obtained from their mothers or caregivers. The study was approved by the University of KwaZulu-Natal Biomedical Research Ethics Committee, Durban, South Africa; the University of the Free State Ethics Committee, Bloemfontein, South Africa; and the Oxford Research Ethics Committee, Oxford, United Kingdom.

## Author contributions

VV and PG wrote the paper; contributed to the study conception and design; and contributed to the acquisition, analysis, and interpretation of the data. NL, EL, VR, AL, HNK, JGP, JG, JC, MML, and AG contributed to the study conception and design and analysis and interpretation of the data. EA, AS, RD, and CT contributed to the acquisition of the data; AG contributed to analysis and interpretation of the data; MM, JR, PK, AJP, GTW, KD, JF, BDW, PJ, and TN contributed to acquisition and interpretation of the data.

## Supplementary Material

Supplemental data

## Figures and Tables

**Table 1:**
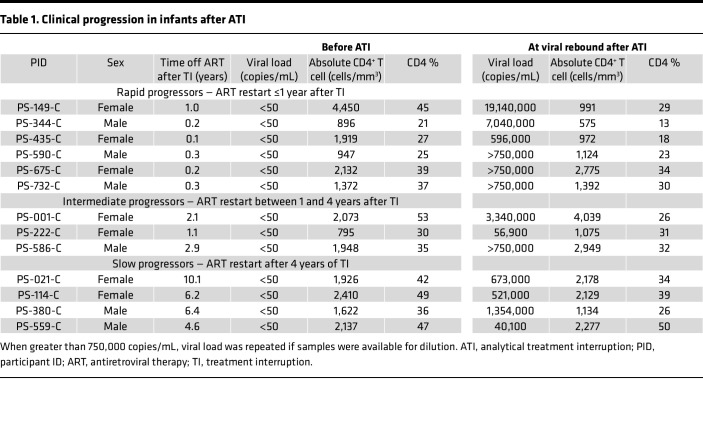
Clinical progression in infants after ATI

## Version 1 01/05/2023

In-Press Preview

## Version 2 02/08/2023

Electronic publication
