# Supplementary material for: Slow progression of pediatric HIV associates with early CD8+ T cell PD-1 expression and a stem-like phenotype
Source: JCI Insight. 2023 Feb 8;8(3):e156049. doi: 10.1172/jci.insight.156049 (PMC9977437; doi:10.1172/jci.insight.156049)
Supplement: Supplemental data [file jciinsight-8-156049-s195.pdf]

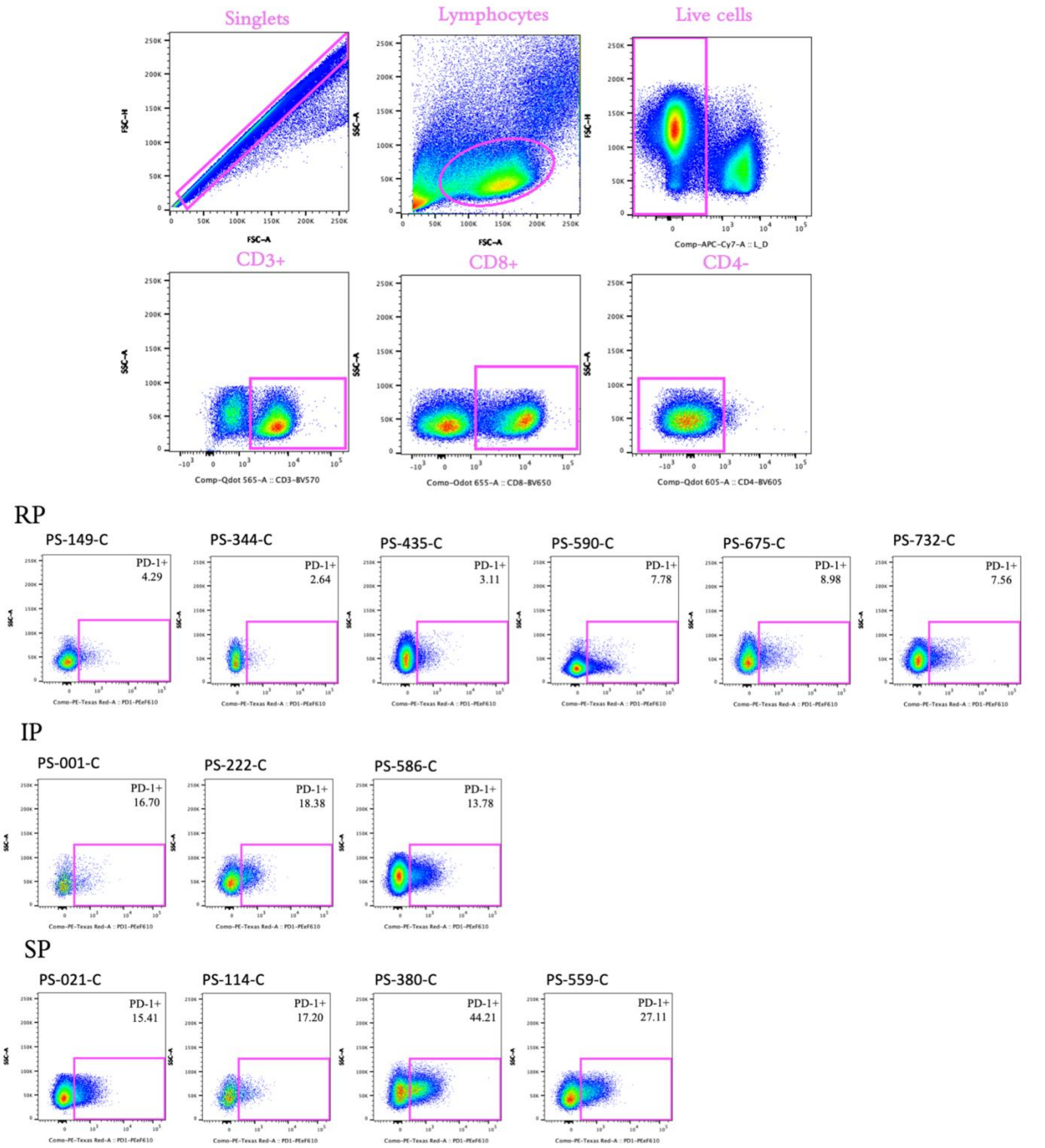

Supplemental Figure 1. Strategy analysis to gate on PD1+ CD8+ T-cells and individual PD-1+ gates.

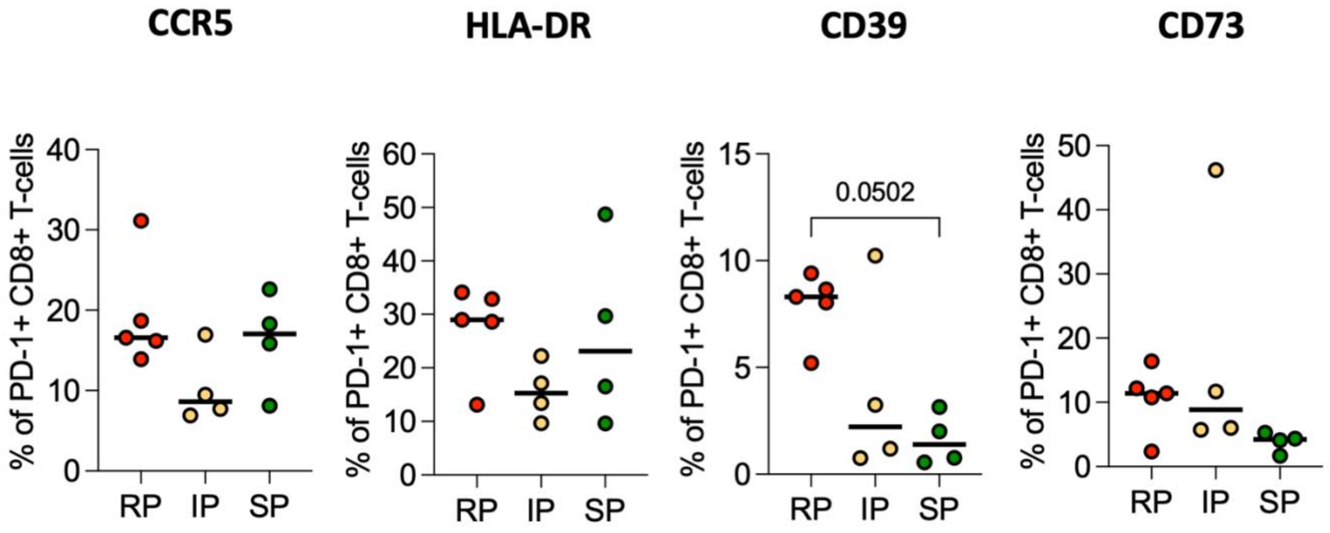

Supplemental Figure 2. Co-expression of CCR5, HLA-DR, CD39 and CD73 on PD-1+ CD8+ T-cells in RP, IP and SP. Statistical comparison between three and four groups was done using Kruskal-Wallis' test followed by Dunn's test to correct for multiple comparisons.

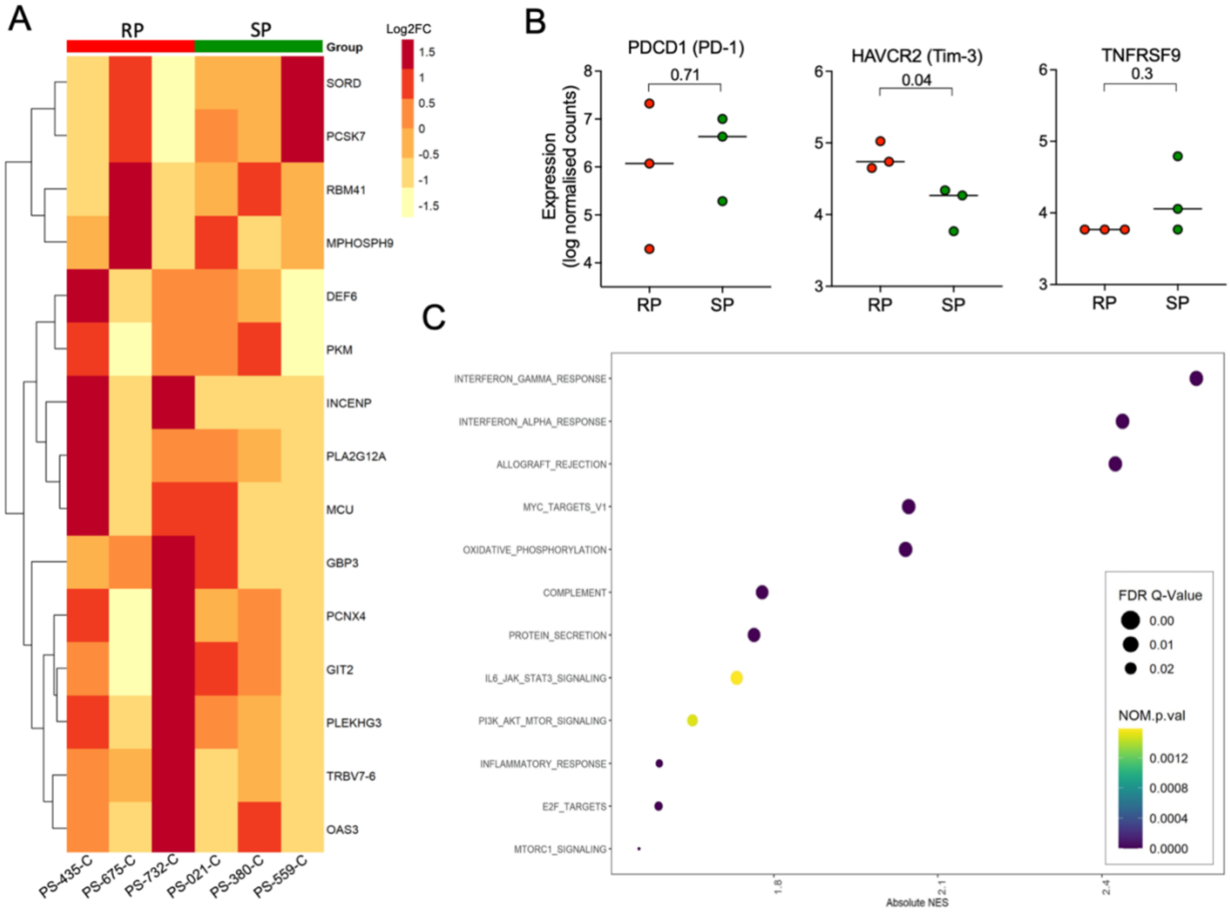

Supplemental Figure 3. Transcriptional analysis shows increased expression of Tim-3 gene (HAVCR2) in PD-1^{HIGH} CD8+ T cells from RP. A. Heatmap is showing the differential gene expression (DGE) in sorted PD-1high CD8+ T-cell population of three SPs and RPs. B. Scaled log-normalized expression values of genes of interest showing similar *PD-1* expression and higher expression of *HAVCR2* (Tim-3) on RP. C. Pathway enrichment for genes significantly associated with SPs. Significant gene sets were filtered based on p-adjusted value less than 0.05.

Supplemental Table 1. Clinical data of infants after ATI at the ELISPOT timepoint analysis.

PID	Absolute CD4+ T cell (cells/mm^3)	CD4 %	Viral load (copies/mL)*
Rapid progressors (RP) - ART restart ≤ 1 year after TI			
PS-149-C	1285	15	2,740,000
PS-344-C	581	13	210,000
PS-675-C	1570	14	714,000
PS-732-C	1032	17	657,000
Intermediate progressors (IP) - ART restart between 1-4 years after TI			
PS-001-C	1965	31	170,000
PS-222-C	993	25	484,000
PS-586-C	2155	29	>750,000
Slow progressors (SP) - ART restart after 4 years of TI			
PS-021-C	2179	33	224,639
PS-114-C	1699	37	276,000
PS-380-C	1567	28	>750,000
PS-559-C	1140	24	699,000

* When >750,000 copies/mL, viral load was repeated if samples were available for dilution

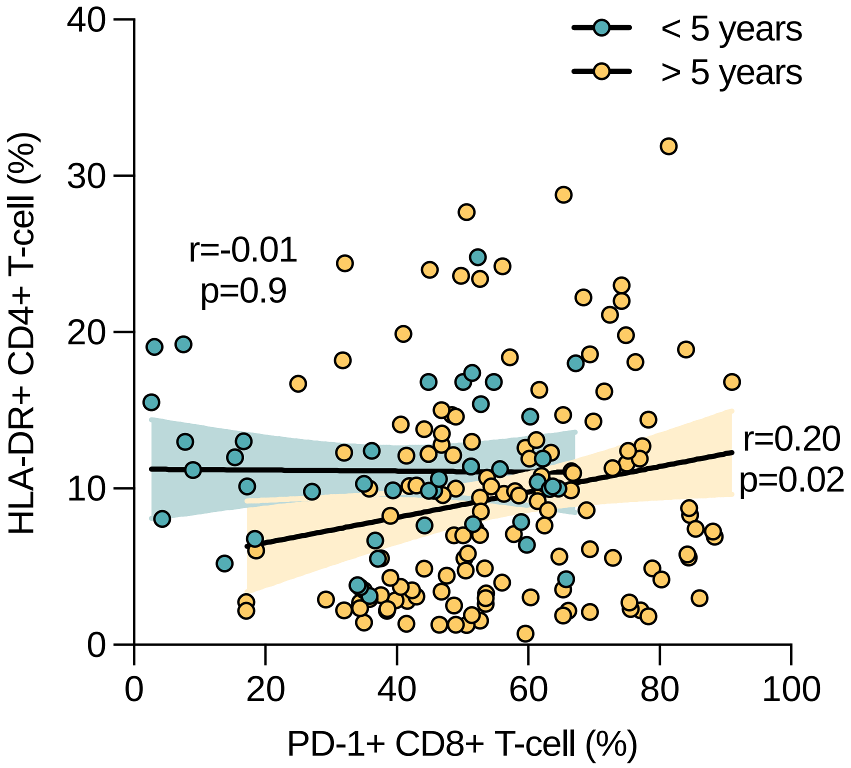

Supplemental Figure 4. PD-1+ CD8+ T-cell correlation with immune activation (HLA-DR+) in HIV-infected children before and after 5 years of age. Spearman rank tests were used for correlations. The best-fit line and 95% confidence bands are shown.

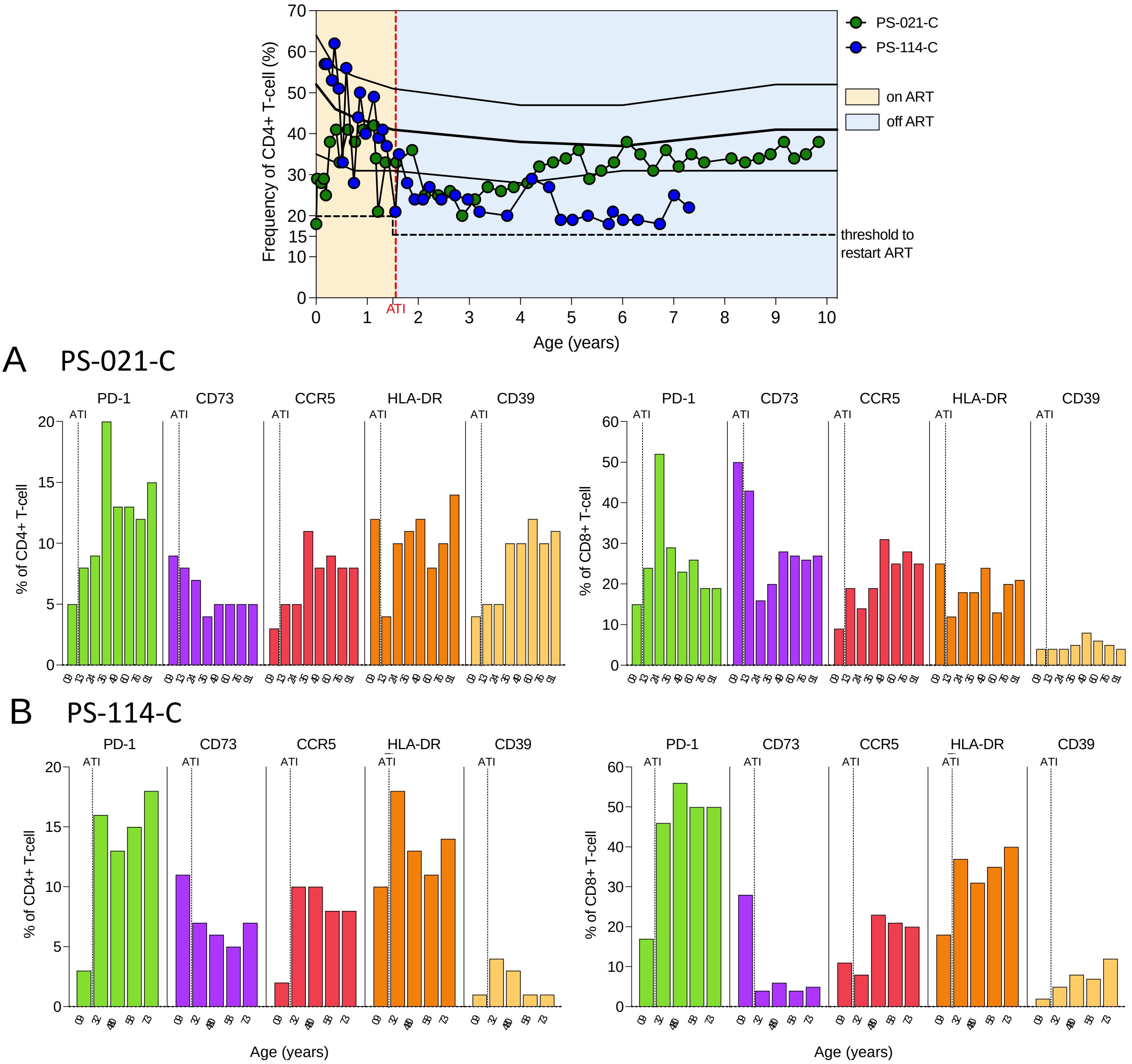

Supplemental Figure 5. Longitudinal T-cell immunophenotype of two SPs. PS-021-C (green dots) maintained CD4+ T-cell within the normal range while PS-114-C (blue dots) experienced a decline of CD4+ T-cell after ATI and near the threshold to re-start ART (black dotted line). The 10th, 50th and 90th percentiles for HIV-uninfected children are represented by the three black lines. The expression of each marker on CD4+ and CD8+ T-cell are shown before and after ATI for PS-021-C (A) and PS-114-C (B).

Supplemental Table 2. Clinical data of paediatric and adult groups.

	HIV-Exposed Uninfected (HEU) (n=16)	Paediatric Slow Progressors (PSP) (n=18)	Paediatric Progressors (PP) (n=18)	Chronic Viraemic Adults (VA) (n=18)
**Age (years)**	13.8 [8.3 - 16.9]	12.1 [10.9 - 15.2]	13.35 [11.9 - 16.8]	29 [26 - 35.5]
**Sex**	9M:7F	8M:10F	9M:9F	9M:9F
**Plasma HIV RNA (copies/mL)**	-	18,000 [980 - 120,000]	165,000 [43,500 - 590,000]	24,883 [17,128 - 196,563]
**Absolute CD4+ T-cell (cell/mm^3)**	-	750 [665 - 917]	246 [102 - 306]	411 [388 - 515]
**Relative CD4+ T-cell (%)**	-	31 [25 - 34]	9 [3 - 16.5]	26 [19 - 37]
**CD4:CD8 ratio**	-	0.71 [0.44 - 1.10]	0.19 [0.10 - 0.36]	0.40 [0.27 - 0.73]

*Data shown in median and interquartile

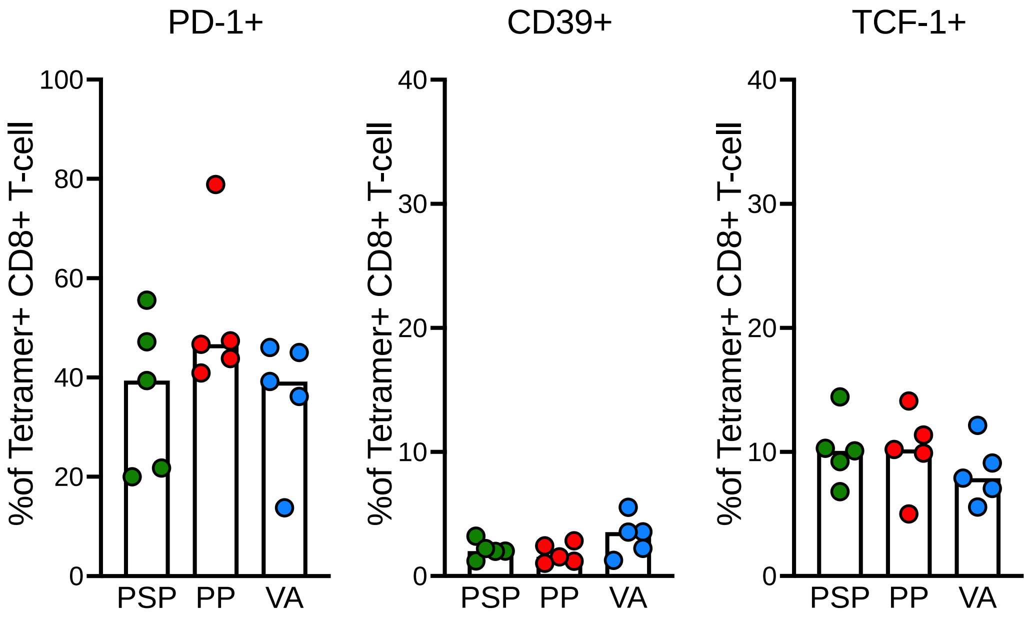

Supplemental Figure 6. Frequency of PD-1+, CD39+, and TCF-1+ on pp65(CMV)-Tetramer+ CD8+ T-cell for each group. Statistical comparison between three and four groups was done using Kruskal-Wallis' test followed by Dunn's test to correct for multiple comparisons.

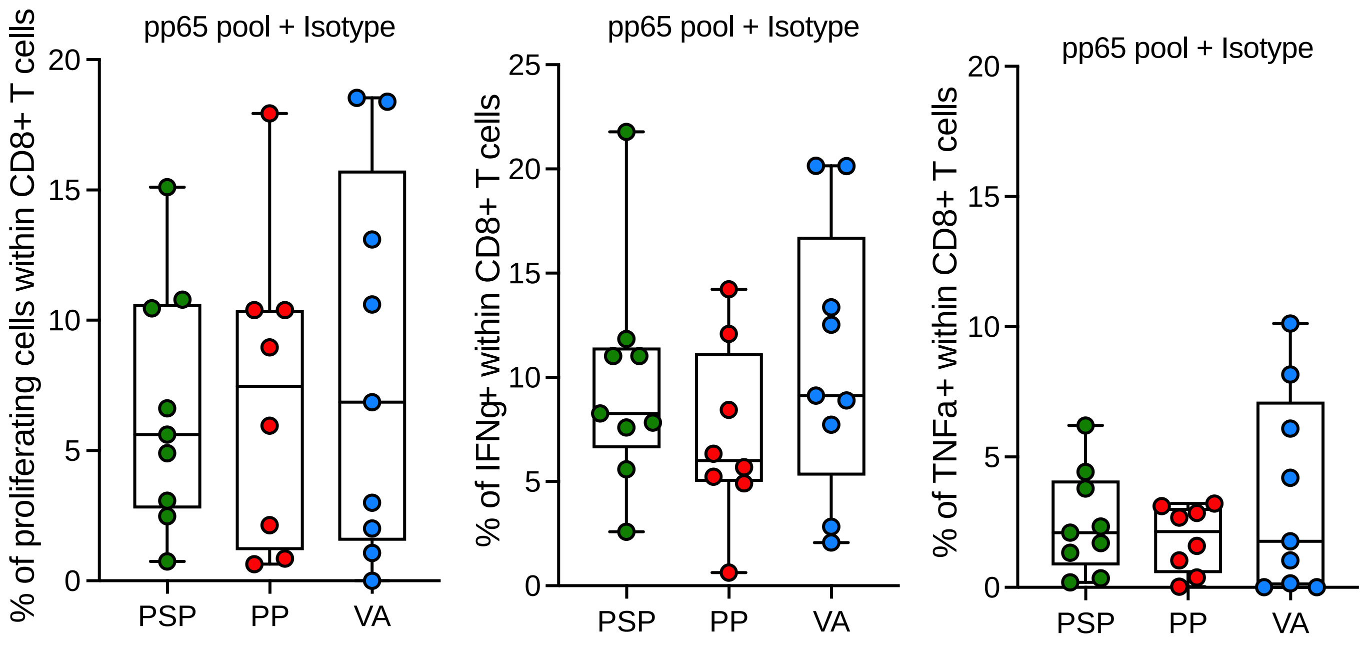

Supplemental Figure 7. Frequency of total, IFN-γ+ and TNF-α+ proliferated CD8+ T-cell after seven days of stimulation with pp65-pool. Statistical comparison between three and four groups was done using Kruskal-Wallis' test followed by Dunn's test to correct for multiple comparisons.

Supplemental Table 3. FACS panels.

A. Antibodies used for T-cell immunophenotype of the ATI study.

Antibody	Fluorochrome	Clone	Company	Dilution
**CD3**	BV605	UCHT1	Biolegend	1:50
**CD4**	BV650	RPA-T4	Biolegend	1:50
**CD8α**	BV570	RPA-T8	Biolegend	1:50
**CD45RA**	AlexaFluor700	H100	Biolegend	1:50
**CCR7**	Pacific Blue	G043H7	Biolegend	1:50
**CCR5**	PE-Cy7	HM-CCR5	Biolegend	1:100
**HLA-DR**	APC-R700	G46-6	BD	1:100
**CD39**	APC	A1	eBioscience	1:50
**CD73**	PE	AD-2	Biolegend	1:200
**PD-1**	PE-eFluor610	J105	eBioscience	1:50
**Viability**	near-IR	N/A	Invitrogen	1:50 (of 1:200 stock)

B. Antibodies used for PD-1+ CD8+ T-cell immunophenotype.

Antibody	Fluorochrome	Clone	Company	Dilution
**CD3**	PE-Cy5	UCHT1	Biolegend	1:50
**CD4**	BV650	RPA-T4	Biolegend	1:50
**CD8α**	PE-Cy7	RPA-T8	Biolegend	1:50
**CD45RA**	BV605	H100	Biolegend	1:50
**CCR7**	Pacific Blue	G043H7	Biolegend	1:50
**CD27**	BV510	M-T271	Biolegend	1:100
**CXCR5**	AlexaFluor700	J252D4	Biolegend	1:50
**CD127**	AlexaFluor647	HIL-7R-M21	BD	1:10
**CD39**	APC	A1	eBioscience	1:50
**TCF-1***	PE	7F11A10	Biolegend	1:50
**PD-1**	PE-eFluor610	J105	eBioscience	1:50
**Viability**	near-IR	N/A	Invitrogen	1:50 (of 1:200 stock)

*Intracellular

C. Antibodies used for Tetramer+CD8+ T-cell immunophenotype.

Antibody	Fluorochrome	Clone	Company	Dilution
**CD3**	BV711	UCHT1	Biolegend	1:50
**CD4**	BV650	RPA-T4	Biolegend	1:50
**CD8α**	BV570	RPA-T8	Biolegend	1:50
**CD45RA**	AlexaFluor700	H100	Biolegend	1:50
**CCR7**	PerCP-Cy5.5	G043H7	Biolegend	1:50
**CD27**	BV510	M-T271	Biolegend	1:100
**Tetramers**	Pacific Blue		ImmunAware	1:2.5
**CD127**	BV605	A019D5	Biolegend	1:25
**CD39**	APC	A1	eBioscience	1:50
**TCF-1***	PE	7F11A10	Biolegend	1:50
**PD-1**	PE-eFluor610	J105	eBioscience	1:50
**T-bet***	PE-Cy7	4B10	Biolegend	1:25
**Granzyme B**	FITC	GB11	Biolegend	1:50
**Viability**	near-IR	N/A	Invitrogen	1:50 (of 1:200 stock)

*Intracellular

D. Antibodies used for proliferation assay.

Antibody	Fluorochrome	Clone	Company	Dilution
**CD3**	PE-Cy5	UCHT1	Biolegend	1:50
**CD4**	BV605	RPA-T4	Biolegend	1:50
**CD8α**	BV570	RPA-T8	Biolegend	1:50
**IFN-γ***	AlexaFluor700	B27	Biolegend	1:50
**TNF- α***	FITC	MAb11	Biolegend	1:50
**CD107a**	BV650	H4A3	Biolegend	1:25
**CTV**	Pacific Blue	N/A	Life Technologies	1:2.5
**CD127**	PE-Cy7	HIL-7R-M21	BD	1:25
**CD39**	APC	A1	eBioscience	1:50
**TCF-1***	PE	7F11A10	Biolegend	1:50
**PD-1**	PE-eFluor610	J105	eBioscience	1:50
**Viability**	near-IR	N/A	Invitrogen	1:50 (of 1:200 stock)

*Intracellular
